# N-Acetyl Cysteine Inhibits Endothelin-1-Induced ROS Dependent Cardiac Hypertrophy through Superoxide Dismutase Regulation

**DOI:** 10.22074/cellj.2016.3746

**Published:** 2015-07-11

**Authors:** Sobia Mushtaq, Tahir Ali, Qamar Javed, Sobia Tabassum, Iram Murtaza

**Affiliations:** 1 Department of Biochemistry, Quaid-i-Azam University, Islamabad, 45320, Pakistan; 2 Department of Biotechnology, International Islamic University, Islamabad, Pakistan

**Keywords:** Cardiac Hypertrophy, Endothelin-1, Oxidative Stress, Superoxide Dismutase, Reactive Oxygen Species

## Abstract

**Objective:**

Oxidative stress down regulates antioxidant enzymes including superoxide dismutase (SOD) and contributes to the development of cardiac hypertrophy. N-Acetyl cysteine (NAC) can enhance the SOD activity, so the aim of this study is to highlight the inhibitory role of NAC against endothelin-1 (ET-1)-induced cardiac hypertrophy.

**Materials and Methods:**

In this experimental study at QAU from January, 2013 to March, 2013. ET-1 (50 µg/kg) and NAC (50 mg/kg) were given intraperitoneally to 6-day old neonatal rats in combination or alone. All rats were sacrificed 15 days after the final injection. Histological analysis was carried out to observe the effects caused by both drugs. Reactive oxygen species (ROS) analysis and SOD assay were also carried out. Expression level of hyper- trophic marker, brain natriuretic peptide (BNP), was detected by western blotting.

**Results:**

Our findings showed that ET-1-induced cardiac hypertrophy leading towards heart failure was due to the imbalance of different parameters including free radical-induced oxidative stress and antioxidative enzymes such as SOD. Furthermore NAC acted as an antioxidant and played inhibitory role against ROS-dependent hypertrophy via regulatory role of SOD as a result of oxidative response associated with hypertrophy.

**Conclusion:**

ET-1-induced hypertrophic response is associated with increased ROS production and decreased SOD level, while NAC plays a role against free radicals-induced oxidative stress via SOD regulation.

## Introduction

Endothelin-1 (ET-1) is a useful vasoconstrictor peptide that is expressed by endothelium and also produced in the heart due to many stresses. ET-1 is considered as one of the neurohumoral factors causing the cardiac hypertrophy. In cultured cardiac myocytes, it induces hypertrophy through G protein-coupled receptors (1). Cardiac hypertrophy is a mechanism associated with the enlargement of cells without proliferation and observed in certain cardiovascular disorders. Even though the initial hypertrophic response may be beneficial, continued hypertrophy results into heart failure (2). Atrial natriuretic peptide (ANP) has been characterized as a cardiac hormone, mainly produced in and released from the atrium in the normal heart (3), while brain natriuretic peptide (BNP), the second member of natriuretic peptide family, is predominantly synthesized in and secreted from ventricle (4-6). Both are elevated in cardiac overload, including cardiac hypertrophy (7, 8).

It is well known that oxidative stress is generated via reactive oxygen species (ROS) that plays an important role in transition from cardiac hypertrophy to heart failure (9). ET-1 plays an important role to increase ROS level in the heart (10, 11). ROS has been proved to be important mediators of ET-1-induced growth-promoting signaling events during hypertrophic pathways in vascular smooth muscle cells (12) and cardiomyocytes (13). The role of ROS that has been further confirmed by ET-1-induced cardiac hypertrophy can be inhibited by pretreatment with antioxidants (14). Lower ROS levels regulate the response of cardiac myocytes to hypertrophic stimuli; however, at later stage of cardiac hypertrophy when ROS levels significantly exceed the capacity of an antioxidant defense system such as superoxide dismutase (SOD), glutathione peroxidase (GPOX) and catalase (CAT), it leads to the myocardial dysfunction and/or injury (9). Increased ROS production is associated with contractile dysfunction in heart failure, ET-1 increases ROS production in left ventricle that is inhibited by nicotinamide adenine dinucleotide phosphate (NADPH) oxidase inhibitor apocynin (15).

Antioxidants such as N-acetylcysteine (NAC) have been used to identify the role of ROS in various biological and pathological processes. NAC plays an essential role to normalize the oxidative stress-mediated overexpression of myocardial protein kinase Cβ2 (PKCβ2) and connective tissue growth factor (CTGF) that is followed by attenuating development of myocardial hypertrophy. Recently it has been reported that NAC enhances the activity of tissue specific antioxidants such as SOD (16, 17). Mitochondrial, cytosolic as well as extracellular SODs are enzymes that have a potential role in ROS regulation by scavenging superoxide anions (18). Our present study aimed to investigate the inhibitory role of NAC through SOD regulatory effect in ET-1-induced cardiac hypertrophy.

## Materials and Methods

### Drugs and chemicals

In this experimental study at QAU from January, 2013 to March, 2013, ET-1 and NAC were purchased from Sigma Aldrich (St. Louis, MO, USA). BNP antibodies, goat anti-rabbit IgG-AP antibody and 0.45-μm pore-size nitrocellulose membrane were purchased from Santa Cruz Biotechnology (Dallas, Texas, USA). Alkaline phosphatase (AP), 5-bromo- 4-chloro-3-indolyl-phosphate (BCIP) and nitro blue tetrazolium (NBT) were purchased from Tiangen (Beijing, China). Sodium acetate, N-diethyl-peraphenylenediamine (DEPPD), ferrous sulphate, NaCl, KH_2_PO_4_, Na_2_HPO_4_, KCl, L-methionine, triton X-100, riboflavin were purchased from Merck Chemicals (Germany).

### Establishment of animal model for cardiac hypertrophy

The experimental animal were maintained and cared based on the National Institute of Health (NIH) guidelines for the human use of laboratory animal models, and the Ethics Committee of Quaid-i-Azam University confirmed the study for animal model handling. Neonatal Sprague-Dawley rats (n=20) received daily intraperitoneal injections of ET-1 (50 μg/kg) and NAC (50 mg/kg) on postnatal days 5-9. These experiments were performed in triplicate. The controls received an equal volume of 0.9% NaCl as described previously (3). Rats were separated into four groups with same number of rats in each group. Group-1 received injections of ET-1, group-2 received ET-1 in combination with NAC, group-3 received NAC alone and group-4 served as control group. All of them were sacrificed on postnatal day 24 (15 days after the final injection). Hearts were removed and stored at -80˚C after treatment with liquid nitrogen. Blood was collected and centrifuged at 4000 rpm for 10 minutes to separate serum and stored at -20˚C. Five samples were collected from each group.

### Reactive oxygen species analysis of serum samples

ROS detection for standard curve formation was assayed according to the method of Hayashi et al. (19). In 0.1 M sodium acetate buffer (pH=4.8), DEPPD (R1) was dissolved to get final concentration of 100 μg/ml and ferrous sulfate (R2) was dissolved in sodium acetate buffer to achieve final concentration of 4.37 μM R1 and R2 were mixed in a ratio of 1:25 to make analysis solution. This solution was then added as starter in a cuvette (3 ml) followed by the addition of sodium acetate buffer and hydrogen peroxide (H_2_O_2_) in order to be used as positive control, while the absorbance of serum samples was measured at 505 nm using Agilent 8453 ultravoilet-visible (UV) Spectrophotometer (Agilent Tech., UK).

### Superoxide dismutase assay of serum samples

SOD assay of samples was carried out using the modified method of Beyer and Fridovich (20). Reaction mixture was prepared with phosphate buffer saline (PBS) (NaCl, KH_2_PO_4_, Na_2_HPO_4_ and KCl), L-methionine, NBT and triton X-100 followed by the addition of serum sample. After illumination with fluorescent lamp, riboflavin was added to initiate the reaction. Sample mixture was then delivered into cuvettes and absorbance was measured at 560 nm. Control serum was also analyzed in parallel.

### Histological analysis

Hearts excised from animal model were fixed in 10% formalin, embedded in paraffin, sectioned horizontally into 7-μm-thick slices, and stained with hematoxylin-eosin (HE) using the method of Fischer et al. (21). Polarized light and bright field microscopes were used to visualize sections and images were then taken with similar settings.

### Detection of hypertrophy marker by western blotting

Serum samples were used for detection of BNP by western blot. These samples were subjected to sodium dodecyl sulfate polyacrylamide gel electrophoresis (SDS-PAGE) method and transferred onto 0.45-μm pore-size nitrocellulose membrane at 100 V for 2 hours in transfer buffer (25 mM Tris, 192 mM glycine and 20% methyl alcohol at pH=8.3) according to the manufacturer’s instructions. After being blocked with nonfat milk, the membranes were incubated with primary antibodies with dilution of 1:500 overnight at 4˚C and then with goat anti-rabbit IgG-AP antibody with dilution of 1:2,000 for one hour at room temperature. The membranes were developed for 30 minutes in AP color development substrate solution containing 1ml of 1X AP reaction buffer.

### Statistical analysis

Data were analyzed by the SPSS (SPSS Inc., USA) version 16 and presented as means ± standard deviation (SD). One way ANOVA followed by Tukey’s multiple comparison test was used to evaluate significant difference among the groups. ROS and SOD data were compared by histograms. A P value <0.05 was considered statistically significant.

## Results

### Endothelin-1-induced cardiac hypertrophic changes in neonatal rat heart

**Fig.1:**
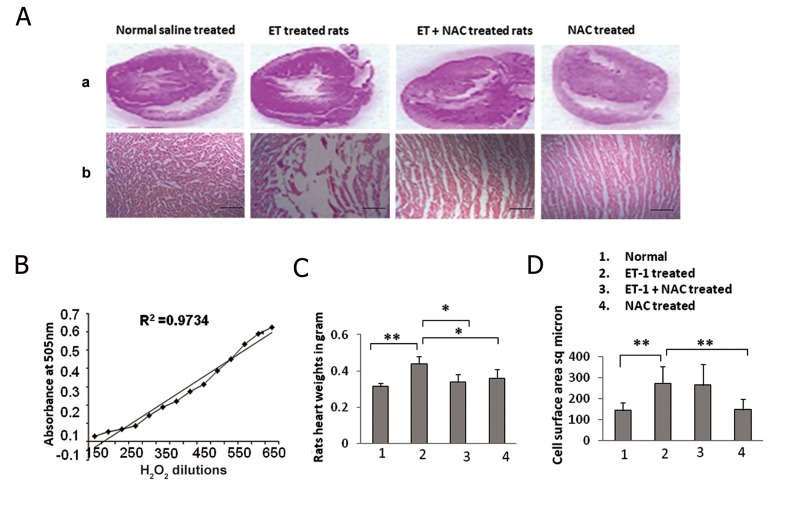
A. Histological analysis of heart tissues with hematoxylin-eosin staining (magnification ×40); horizontal sections; cross sections (scale bar=100 μm), B. Calibration curve for ROS analysis. C. Rats heart size of respective groups (n=20) and D. Cell surface area measurement in respective groups analyzed by measuring 200 cells in 40 to 50 fields. *; P≤0.05, **; P≤0.01, ET-1; Endothelin-1, NAC; N-acetylcysteine and ROS; Reactive oxygen species.

**Fig.2:**
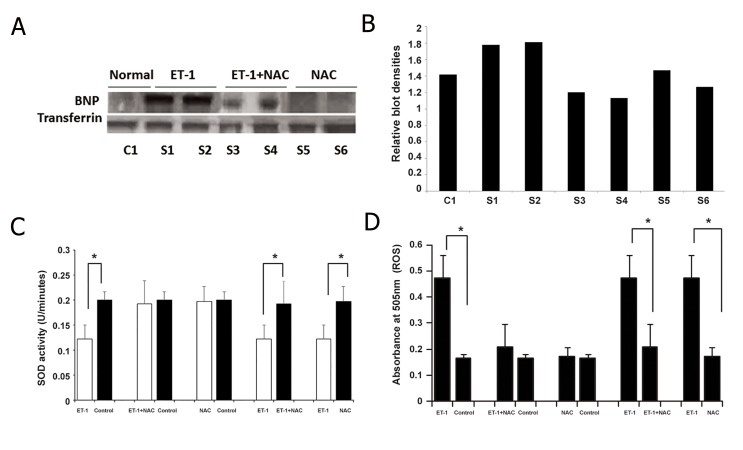
A. Western blot expression of serum samples in normal, ET-1 and NAC treated neonatal rats with BNP as hypertrophic marker compared to loading control transferrin, B. Densitometric quantification of immunoblot bands using software Image J, C. Comparison of the serum SOD (U/mL) activity in ET-1, ET-1+NAC, NAC treated and normal rats. *; P≤0.05 and D. Comparison of the serum ROS levels in ET-1, ET-1+NAC, NAC treated and normal rats. *; P≤0.05, ET-1; Endothelin-1, NAC; N-Acetyl cysteine, BNP; Brain natriuretic peptide, SOD; Superoxide dismutase and ROS; Reactive oxygen species.

Present study used BNP as a hypertrophic marker to confirm the effect of ET-1 and NAC in experimental model of hypertrophy with transferrin as a loading control. In the current study, ET-1 effects on neonatal rat heart including BNP expression and histology of heart tissues were examined. Hearts were hypertrophied after ET-1 treatment. Under light microscope (Dialux 20EB, Canada), sections were studied at ×40 magnifications. Cell surface areas were measured by using software SPOT cam w 4.0 (Computer assisted modeling). About 100-200 cardiomyocytes were examined in 20-50 fields. Amplitudes of cross section areas were also measured with scale bar at 100 μm (Fig.1A). Calibration curve of H_2_O_2_ was constructed to measure and correlate the ROS (Fig.1B). As shown in figure 1C and D, the heart size and the cell surface area of the myocytes were increased in ET-1-treated rats compared to the normal subjects. In experimental animal models serum, an increase in the expression of BNP, a cardiac hypertrophy marker, was observed (Fig.2A). This study was carried out with five numbers of replicates in each group. Densitometric quantification of immunoblots was also performed using software Image J (Fig.2B).

### Regulatory role of endothelin-1 in superoxide dismutase and reactive oxygen species levels in cardiac hypertrophic condition

In the present investigation, the free radical status was studied in ET-1-treated neonatal rat model. Figure 2C and D shows serum ROS and SOD levels were significantly up and down regulated in response to ET-1 treatment, respectively. An increase of free radical production and a decrease of antioxidant levels including SOD induce oxidative stress. Oxidative stress acts as a main player in cardiac hypertrophy. Present data suggests ET-1 induced oxidative stress via ROS and SOD-regulation in cardiac hypertrophy.

### Inhibitory role of N-acetylcysteine in endothelin-1-induced cardiac hypertrophic changes via superoxide dismutase and reactive oxygen species regulation

NAC has been previously reported as an antioxidant. In the present study, NAC was administrated along with ET-1 to animal models. The myocytes surface areas were relatively decreased in NAC+ET-1 treated group when compared to the ET-1 treated group (Fig.1D). The serum SOD level was significantly increased after NAC treatment, suggesting a possible link of SOD and NAC. Serum ROS levels were significantly decreased in ET-1+NAC treated group compared to the ET-1 treated group. Thus the results suggest that ET-1 stimulated the hypertrophy signals in the heart and NAC contributed to the inhibition of ET-1-stimulated hypertrophy signals via ROS and SOD regulation.

## Discussion

Cardiac hypertrophy is an adaptive response to a chronic increase in workload on the heart, which can progress into a state of heart failure. At cellular level, the hypertrophy is characterized by increase in cell size along the expression of specific protein markers like BNP. It has been previously documented that ET-1 is involved in the heart failure by stimulating cardiac hypertrophy signals (22-27). In the present study, enlargement disarray of myocytes, increased interstitial fibrosis and the elevated expression of BNP in ET-1 treated group were observed.

It has been well established that oxidative stress generated by elevated ROS participates in the cardiac hypertrophy and heart failure (28). Increased oxidative stress helps in the stimulation of redox-sensitive signaling pathways which leads to fibrosis, cardiac remodeling and cardiac hypertrophy. Current results of enhanced serum ROS level in the ET-1 treated animal models suggest that ET-1 induced hypertrophic response via free radical regulation.

In present findings, NAC treatment conferred protection against ET-1-stimulated response by diminishing the indirect ROS generation via SOD regulation. The serum SOD and ROS levels were up and down regulated in ET-1+NAC treated group, respectively, compared to the ET-1 treated group. SOD as an antioxidant is the first line of defense against ROS (29) and NAC treatment significantly improves antioxidants (including SOD) regulatory mechanism of a body (30, 31). It has been also reported that NAC as an antioxidants could inhibit the NADPH oxidase activation, which is considered as one source of ROS (29). It shows that NAC treatment indirectly inhibits the ROS-induced changes via antioxidant regulation in diseases condition including cardiac hypertrophy.

## Conclusion

Present study demonstrated that the development of oxidative stress via free radicals can be counterbalanced by antioxidants and it plays an important role in cardiac hypertrophy. NAC acting as an effective scavenger of free radicals also contributes to the maintenance of the cellular antioxidant metabolism. Our hypothesis confirmed that the application of NAC treatment reduces the ET-1-induced cardiac hypertrophy and improved cellular relaxation subsequent to the prevention of elevated oxidative stress induced by free radicals. Thus current study suggests that use of specific antioxidants may be helpful in future therapy of cardiac hypertrophy leading to heart failure.
